# The cost effectiveness of vaccinating against Lyme disease.

**DOI:** 10.3201/eid0503.990302

**Published:** 1999

**Authors:** M I Meltzer, D T Dennis, K A Orloski

**Affiliations:** Centers for Disease Control and Prevention, Atlanta, Georgia 30333, USA. qzm4@cdc.gov

## Abstract

To determine the cost effectiveness of vaccinating against Lyme disease, we used a decision tree to examine the impact on society of six key components. The main measure of outcome was the cost per case averted. Assuming a 0.80 probability of diagnosing and treating early Lyme disease, a 0.005 probability of contracting Lyme disease, and a vaccination cost of $50 per year, the mean cost of vaccination per case averted was $4,466. When we increased the probability of contracting Lyme disease to 0.03 and the cost of vaccination to $100 per year, the mean net savings per case averted was $3,377. Since few communities have average annual incidences of Lyme disease >0. 005, economic benefits will be greatest when vaccination is used on the basis of individual risk, specifically, in persons whose probability of contracting Lyme disease is >0.01.


### The Cost Effectiveness of Vaccinating against Lyme Disease

**Martin I. Meltzer, David T. Dennis, and Kathleen A. Orloski**
Centers for Disease Control and Prevention, Atlanta, Georgia, USA

To determine the cost effectiveness of vaccinating against Lyme disease, we used a decision tree to examine the impact on society of six key components. The main measure of outcome was the cost per case averted. Assuming a 0.80 probability of diagnosing and treating early Lyme disease, a 0.005 probability of contracting Lyme disease, and a vaccination cost of $50 per year, the mean cost of vaccination per case averted was $4,466. When we increased the probability of contracting Lyme disease to 0.03 and the cost of vaccination to $100 per year, the mean net savings per case averted was $3,377. Since few communities have average annual incidences of Lyme disease >0.005, economic benefits will be greatest when vaccination is used on the basis of individual risk, specifically, in persons whose probability of contracting Lyme disease is $\geq$0.01.

Lyme disease, caused by infection with *Borrelia burgdorferi*, is the most common tick-borne disease in the United States and Europe (1-3). In the United States, the disease has spread slowly, and the number of cases in disease-endemic areas has increased (4-6). Most Lyme disease patients become infected with *B. burgdorferi* near their homes, while engaged in property maintenance, recreation, and relaxation (7). Occupational and recreational activities away from home may also pose a risk (8). Lyme disease prevention based primarily on avoidance of tick bites, use of repellants, early detection and removal of attached ticks, and tick control has not substantially reduced disease incidence (4-6). Therefore, preventive vaccines have been of considerable interest. Results of randomized and blinded phase-III field trials with recombinant *B. burgdorferi* outer surface protein A (rOspA) vaccines indicate that they are safe and efficacious (9,10). On December 21, 1998, the U.S. Food and Drug Administration licensed one of the vaccines (LYMErix, SmithKline Beecham Biologicals, Reixensart, Belgium) for use in the United States (11).

We present the results of an analytic model that evaluates the cost effectiveness of using a vaccine to protect against Lyme disease in the United States.

Address for correspondence: Martin I. Meltzer, National Center for Infectious Diseases, Centers for Disease Control and Prevention, 1600 Clifton Road, Mailstop C12, Atlanta, GA 30333, USA; fax: 404-639-3039; e-mail: qzm4@cdc.gov.

### The Model

Using a computer-based spreadsheet (Excel 5.0 for Windows, Microsoft), we constructed a decision tree (12) to evaluate the cost per case averted (cost effectiveness) to society of vaccinating against Lyme disease (Figure 1). Many data needed to determine the cost effectiveness of vaccinating against Lyme disease are unvalidated, unavailable, or available only from very small databases. Thus, rather than calculate a single estimate of cost per case averted, we examined the effect of combinations of six inputs: cost of vaccination; annual probability of contracting Lyme disease; costs of successfully treating either early symptoms of Lyme disease or one of three sequelae (cardiovascular, neurologic, arthritic); probability of diagnosing and treating early symptoms; probability of sequelae due to early infection; probability of sequelae due to late, disseminated infection.

Mathematically, we examined the effect of altering the values of the inputs by using specialized computer software (@Risk, Palisade Corp., Newfield, NY) (13) that employs Monte Carlo methods (14-16). To use these methods, the researcher defines probability distributions for selected inputs by using available data and

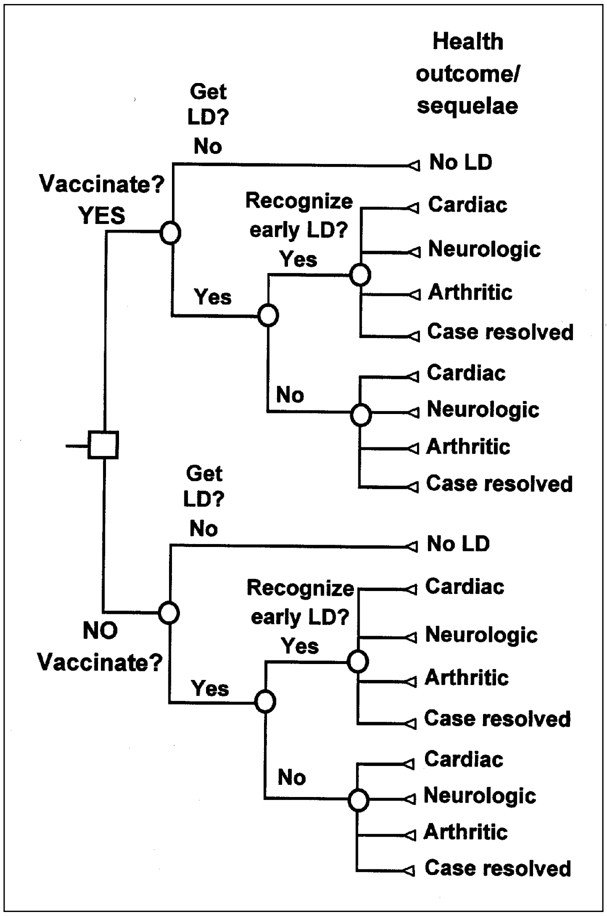

Figure 1: Decision tree to model the cost effectiveness of vaccinating a person against Lyme disease.

enters them into the computer program. For each iteration of the program, an algorithm selects input values from the probability distributions, calculates the net result (here, the cost per case averted), and stores that result. After all iterations (typically 1,000 to 5,000) are completed, the program produces a probability-based distribution of the net result, which can then be used to report statistics such as mean, median, and 5th and 95th percentiles.

### Cost Effectiveness Formula

The formula used to calculate the cost per case of Lyme disease averted was as follows:

$$\text{Cost per case averted} = \frac{\text{\$ of vacc+\$of LD with vacc-\$ of LDw/o vacc}}{\text{Prob LD w/o vacc-Prob LD with vacc}}$$

where $ = cost; vacc. = vaccination; LD = Lyme disease; and prob. = probability. The numerator is the cost of vaccination less any savings resulting from the reduced probability of contracting the disease (decreased incidence) due to vaccination. If the vaccine is not 100% effective in preventing Lyme disease (i.e., if the term Prob. LD with vacc. >0), treatment costs may still be incurred after vaccination. The cost of a case of Lyme disease is the weighted average cost of all health outcomes (Figure 1), where the weights are the probabilities of those outcomes (12). The denominator reflects the change in the probability of Lyme disease due to vaccination.

### Vaccine Timeline

Although experiments have shown that a Lyme disease vaccine using rOspA is safe and immunogenic in both animals and humans (17-23), no data have been published concerning the decrease in antibody levels over more than 20 months (9). Phase-III vaccine field trials used a 0-, 1-, and 12-month immunization schedule, and antibody levels dropped almost 10-fold between the month after the second dose and just before the third dose at month 12 (9). The third dose at month 12 boosted antibodies to levels higher than measured at month 2, but these declined by half by month 20 (9). We assumed, therefore, that an annual booster dose would be required and that the cost-effectiveness model would be repeated annually. When calculating annual benefits, however, we included the discounted savings of preventing Lyme disease that may generate multiyear sequelae.

### Lyme Disease Symptoms and Sequelae

The most common symptoms of infection with *B. burgdorferi* can be categorized as early localized disease (stage I); early disseminated disease (stage II); and later-stage sequelae of disseminated infection (stage III) (24). Stages I and II correspond to the branches labeled "Recognize early LD? Yes" in Figure 1, and stage III corresponds to the branches labeled "Recognize early LD? No." Most early symptoms of Lyme disease respond promptly and completely to short courses of oral antibiotics (25-27). Later-stage sequelae, however, may require costly, more prolonged treatment, sometimes repeated courses of treatment using intravenous cephalosporins, and may not be completely eliminated (28).

If a person, vaccinated or unvaccinated, contracts Lyme disease, the model allows for one of four possible categories of outcomes (Figure 1)

(29-31): cardiovascular sequelae (e.g., high-grade atrioventricular blocks); neurologic sequelae (e.g., isolated cranial nerve palsy, meningitis); arthritic or rheumatologic/musculoskeletal sequelae (e.g., episodic oligoarticular arthritis, arthralgia); and case resolved (after a course of an oral antibiotic such as doxycycline) with no further complications.

The disseminated stages of Lyme disease may be manifested weeks to months after infection (24). However, few data concerning the duration of such sequelae are available. One study, for example, involving 38 patients showed that their long-term clinical sequelae lasted a mean of 6.2 years from onset of disease (32). The use of health-care resources, however, by those patients during that time was not reported. We assumed that cardiovascular sequelae would be treated and resolved in an average of 1 year and that late neurologic and arthritic sequelae would both take an average of 11 years to diagnose and satisfactorily treat to full resolution (initial year of diagnosis and treatment plus 10 years of additional treatment). These assignments of average time are arbitrary and longer than any published average, which maximizes estimated economic benefits of using a vaccine.

### Probabilities

We selected three probabilities (0.005, 0.01, and 0.03) of contracting Lyme disease (Table 1) on the basis of data concerning disease incidence in Lyme disease-endemic areas (33-36); the probability of 0.03 is among the highest reported. (Before the risk for Lyme disease was widely recognized, a one-time annual incidence of 10% was reported in a community of 190 people living next to an open nature preserve [37].) Vaccine efficacy in preventing Lyme disease was 50% (95% confidence intervals [CI]: 14% to 71%) after the first two doses and 78% (95% CI: 59% to 88%) after three doses (9,11). We assumed Lyme disease vaccine to be 85% effective, which is near the upper end of the 95% confidence limits and thus maximizes estimated economic benefits. We selected 0.6 to 0.9 as the range of probability of early diagnosis and treatment on the basis of a study on the economic cost of Lyme disease, which included data from an expert panel (38). For the Monte Carlo simulations (14-16), we constructed the distributions describing the probabilities of having one of the three sequelae (due to either early or late disseminated disease) using data from the previously mentioned expert panel (Table 1) (38). The distributions describing cardiac and neurologic complications associated with early Lyme disease are uniform, defined by using minimum and maximum values (39) and reflecting the uncertainty regarding a most likely value (38). All other distributions are triangular (39), with minimum, most likely, and maximum values (Table 1).

Table 1. Probabilities and their statistical distributions

| Item | Values | Type of distribution[a] |
|---|---|---|
| Probability of contracting LD[b] | 0.005, 0.01, 0.03 | Fixed intervals[c] |
| Effectiveness of vaccine | 0.85 | Fixed |
| Probability of early detection of LD | 0.6 - 0.9 | Fixed intervals[cd] |
| Probability of sequelae[e] if detect LD early | | |
| Cardiac | 0 - 0.01 | Uniform[f] |
| Neurologic | 0 - 0.02 | Uniform[f] |
| Arthritic | 0.02-0.05-0.07 | Triangular[g] |
| Case resolved | Residual[h] | N/A |
| Probability of sequelae if do not detect LD early | | |
| Cardiac | 0.02-0.03-0.06 | Triangular |
| Neurologic | 0.02-0.15-0.17 | Triangular |
| Arthritic | 0.5-0.6-0.62 | Triangular |
| Case resolved | Residual[h] | N/A |

[a]Statistical distribution used in Monte Carlo simulations (14-16).
[b]LD = Lyme disease.
[c]Iterations are run by using different combinations of the probabilities of infection and cost of treatment (Table 2).
[d]The interval between the minimum and the maximum is divided into 0.1 increments.
[e]See text for description of sequelae.
[f]Uniform distribution implies that there is an equal chance that any number between, and including, the minimum and maximum will be used for a given iteration.
[g]Triangular distribution is defined by points of minimum, most likely, and maximum.
[h]The probability of an LD case being successfully resolved (i.e., no further sequelae) is 1 - (sum of the probabilities of cardiac + neurologic + arthritic symptoms).

### Vaccination Costs

Although a Lyme disease vaccine has been licensed (11), data are not available on the actual cost of vaccination, which includes costs of the vaccine, its administration, time spent in receiving the vaccine, travel, and treatment of adverse side-effects of vaccination. To allow for variation caused by variables such as location of provider, type of provider, and type of third-party payer, we estimated cost effectiveness by using three costs: $50 per person per year, $100 per person per year, and $200 per person per year.

Few data are available on the costs of treating a case of Lyme disease; only one study (29) has documented the charges in 1989 dollars associated with some sequelae. To adjust charges reported in that study to 1996 prices, we multiplied the charges by a factor of 1.528 (medical care component of the consumer price index) (40). These 1996 prices, however, reflected health-care charges paid by health insurance companies and not necessarily actual economic costs (41,42). Thus, to reflect economic costs, the adjusted prices were multiplied by cost-to-charge factor (the weighted average of the urban and rural hospital cost-to-charge ratios used by the U.S. Federal Health Care Finance Administration [43]) of 0.53. Data describing indirect costs, particularly lost productivity, associated with sequelae were unavailable. We therefore assumed that Lyme disease–related cardiac sequelae would cause 14 days of lost productivity, and neurologic and arthritic sequelae would each cause 21 days of lost productivity per year. Each day of lost productivity was valued at $100 (the average income of a workday [1990 dollars inflated to 1996 values] weighted by the age and sex composition of the U.S. workforce) (44). Because we assumed that late-stage neurologic and arthritic complications may take up to 11 years to completely resolve, the 1-year cost estimates for treating these sequelae were replicated over 11 years and then discounted at 3% to the base year (Table 2).

We also altered the estimate of Magid et al. (29) of charges for resolving a case of Lyme disease without complications by doubling the number of office visits to two ($25 each visit) and allowing for 5 hours of lost productivity ($62) for a total of $161 (Table 2). In comparison, a recent study concerning Lyme disease on the eastern shore of Maryland found the median charge for the diagnosis and treatment of Lyme disease was $199 (45); this figure represents charges, and actual economic costs are likely lower than this amount (41,42).

Table 2: Costs of treating one case of Lyme disease and the sequelae due to early and late disseminated disease

| Item | Cost/ year ($) | Length of treat- ment | Total costs[a] ($) |
|---|---|---|---|
| Case resolved: no sequelae | | | |
| Antibiotics | 14 | | |
| Office visits (2) | 50 | | |
| Laboratory tests | 35 | | |
| 5 hrs lost work time | 62 | | |
| Total | 161 | 2-3 wks | 161 |
| Sequelae[b] due to early and late disseminated disease | | | |
| Cardiac-direct[c] | 5,445 | | |
| Cardiac-indirect[d] | 1,400 | | |
| Cardiac-total | 6,845 | ≤ 1 yr | 6,845 |
| Neurologic-direct[c] | 4,865 | | |
| Neurologic-indirect[d] | 2,100 | | |
| Neurologic-total | 6,965 | 11 yrs | 61,243 |
| Arthritic-direct[c] | 1,804 | | |
| Arthritic-indirect[d] | 2,100 | | |
| Arthritic-total | 3,904 | 11 yrs | 34,354 |

[a]All costs that occur over more than 1 year are discounted at a rate of 3% per year.

[b]See text for description of the sequelae.

[c]Direct costs are for all medical costs and are derived from the 1-year charges reported by Magid et al. (29), inflated to 1996 dollars (factor of 1.528) (40), and then adjusted by a cost-to-charge ratio of 0.53 (43) (see text for details).

[d]Indirect costs are the valuation of lost productivity due to Lyme disease-related illness, with each day lost valued at $100. For cardiac-related sequelae, it was assumed that 14 workdays were lost, and for neurologic and arthritic-related sequelae, it was assumed that 21 workdays were lost each year.

### Sensitivity Analyses

To allow for uncertainty caused by lack of data, we conducted multivariate sensitivity analyses in which we simultaneously altered the assumed effectiveness of the vaccine and the cost of treating sequelae. We altered vaccine effectiveness to either 0.75 or 0.95 (compared with 0.85 in the base case [Table 1]), and we multiplied the total costs for treating sequelae (Table 2) by 0.5 or 1.5. For example, for neurologic sequelae, the latter multiplier is equivalent to increasing the days of lost productivity (indirect costs) from 21 days per year to 31.5 days per year. We set the probability of identifying and successfully treating early Lyme disease at 0.80 and the cost of vaccination at $100 per year. The estimates generated by these sensitivity analyses were compared with those generated using the base costs, with an assumed vaccine effectiveness of 0.85, and with the same assumptions for probability of identifying and treating early Lyme disease and cost of vaccination as used in the sensitivity analyses.

### Findings

Assuming a 0.005 probability of contracting Lyme disease, a 0.80 probability of diagnosing and treating early Lyme disease, and a $50 per year cost of vaccination, the mean cost per case averted was $4,466 (5th percentile = $5,408; 95th percentile = $3,587) (Figure 2). The 5th and 95th percentiles were calculated as part of the Monte Carlo simulations (14-16). To enhance clarity, the 5th and 95th percentiles were not plotted on Figure 2. Increasing the cost of vaccination to $100 per year increased the mean cost per case averted to $16,231 (5th = $17,267; 95th = $15,298) (Figure 2). At a cost of vaccination of $200 per year, the mean cost per case averted was $39,761 (5th = $40,858; 95th = $38,830) (Figure 2).

With a 0.01 probability of contracting Lyme disease and a 0.80 probability of correct diagnosis and treatment of early disease, the mean savings per case averted was $1,416 when the cost of vaccination was $50 per year. Vaccination resulted in a net cost of $4,467 when the cost of vaccination was $100 per year and a net cost of $16,231 when the cost of vaccination was set at $200 per year (Figure 2).

When we set the probability of contracting Lyme disease at 0.03 and used the same probability of diagnosis as before (0.80), the mean savings per case averted was $5,337 when the cost of vaccination was $50 per year and $3,377 when the cost of vaccination was $100 per year. The net cost per case averted was $545 when the cost of vaccination was $200 per year (Figure 2).

When the costs of treating sequelae were reduced by half of base costs, at a 0.01 probability of contracting Lyme disease, the average cost of averting one case was $9,684 when vaccine effectiveness was assumed to be 0.75 and $6,877 when vaccine effectiveness was assumed to be 0.95 (Table 3). These are 117% and 54% higher, respectively, than the costs calculated using the base costs (Table 3).

When the costs of treating sequelae were increased to 1.5 times base costs, the equivalent

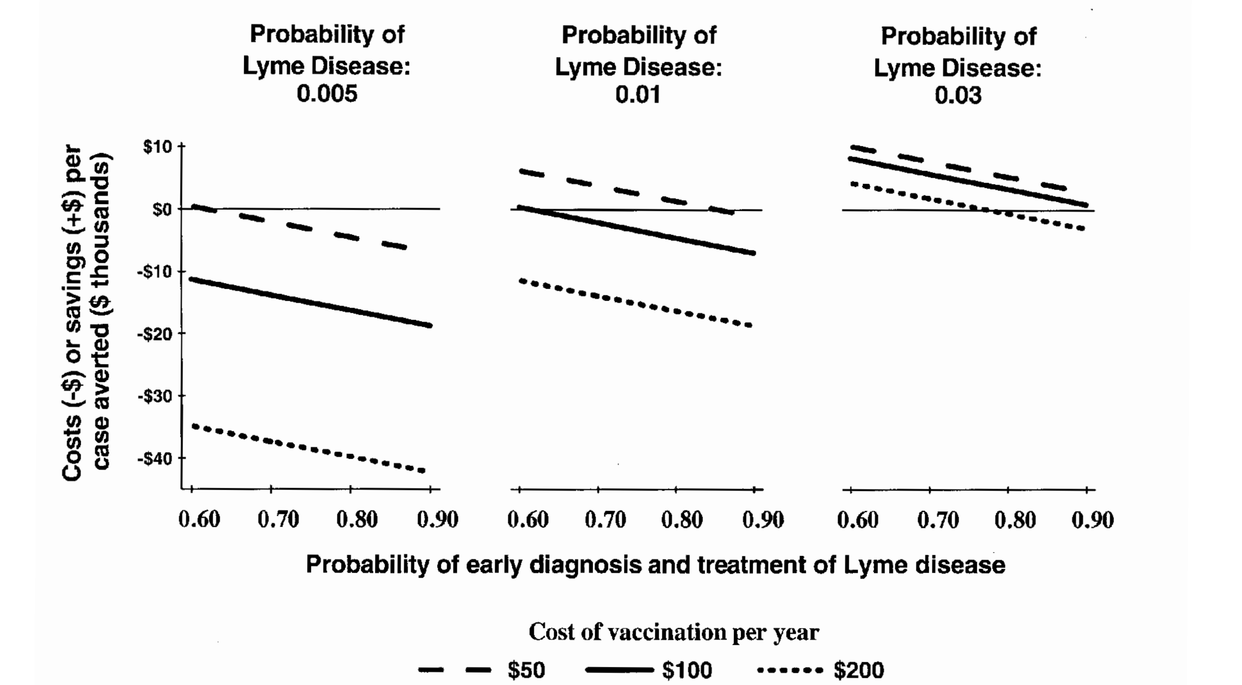

Figure 2: Average cost effectiveness of vaccinating a person against Lyme disease with changes in the cost of vaccination, probabilities of identifying and treating early Lyme disease, and probabilities of contracting Lyme disease. A negative value indicates that vaccinating a person will result in a net cost to society, while a positive value indicates a net savings to society. The results shown are the means from Monte Carlo simulations (see Table 1 and text for further details). Vaccine assumed 85% effective (Table 1).

Table 3. Sensitivity analyses: Cost or savings per case averted (5th, 95th percentiles) by altering assumed vaccine effectiveness and the cost of treating Lyme disease sequelae[a]

| Probability of Lyme disease | Base treatment costs[b] x 0.5 Vaccine effectiveness[d] 0.75 | Base treatment costs[b] x 0.5 Vaccine effectiveness[d] 0.95 | Base treatment costs[c] 0.85 | Base treatment costs[b] x 1.5 Vaccine effectiveness[d] 0.75 | Base treatment costs[b] x 1.5 Vaccine effectiveness[d] 0.95 |
|---|---|---|---|---|---|
| 0.005 | 23,018 | 17,404 | 16,231 | 15,720 | 10,105 |
| | (23,527; 22,556) | (17,947; 16,927) | (17,283; 15,261) | (17,249;14,286) | (11,641;8,703) |
| 0.01 | 9,684 | 6,877 | 4,467 | 2,386 | Net savings[e] |
| | (10,178; 9197) | (7,372; 6,412) | (5,531; 3,487) | (3,846; 958) | (1,220; save[e]) |
| 0.03 | 795 | Net savings[e] | Net savings[e] | Net savings[e] | Net savings[e] |
| | (1,303; 330) | (385; save[e]) | (save; save[e]) | (save;save[e]) | (save; save[e]) |

[a]These results were generated by setting the probability of detecting and successfully treating early Lyme disease at 0.80 and the cost of vaccination at $100 per year.
[b]Base treatment costs are given in Table 2. The data presented in this table were generated by multiplying the costs in Table 2 by either 0.5 (i.e., reducing costs by half) or by 1.5 (i.e., increasing costs by half).
[c]For comparison, the results using the base costs (Table 2) are presented here, assuming a vaccine effectiveness of 0.85. Figure 2 presents the complete set of results using the base costs.
[d]The initial assumed level of vaccine effectiveness was 0.85 (Figure 2).
[e]Net savings are generated when a person is vaccinated against Lyme disease and the costs saved by not having to treat a case of Lyme disease are higher than the costs of vaccination plus the costs of having to treat a case of Lyme disease that occurs after vaccination. The net savings range from $140 (probability of Lyme disease = 0.03, vaccine effectiveness = 0.95, cost of treating Lyme disease sequelae = 0.5 x base costs) to $7,438 (probability of Lyme disease = 0.03, vaccine effectiveness = 0.95, cost of treating Lyme disease sequelae =1.5 x base costs). Note also that in some instances where mean net savings are calculated, the 5th percentiles are net costs.

cost per case averted was $2,386 at a vaccine effectiveness of 0.75, while a vaccine effectiveness of 0.95 was estimated to generate cost savings (Table 3). The former estimate represents a 47% decrease in cost per case averted compared with the base case (Table 3).

These results show that, as the weighted average cost of treating a case of Lyme disease decreases (increases), the cost per case averted through vaccination increases (decreases). An inspection of the formula to calculate the cost per case of Lyme disease averted, presented in the Model section, shows that, as the term $ of LD w/out vacc (in the numerator) decreases, the cost per case averted must increase.

### Conclusions

Because of either lack of data or wide variability in some key variables (e.g., cost of vaccination, risk for Lyme disease), a single answer regarding the cost effectiveness of vaccinating a person against Lyme disease cannot be calculated. The methods we used allow physicians, health-care decision makers, and public health authorities to use Figure 2 and Table 3 to determine the cost effectiveness of vaccination for their specific situations. This simple model can be rerun to provide estimates per case averted for situations not covered in the results presented (e.g., lower or higher probabilities of Lyme disease). The estimates do not include any valuation of a person's willingness to pay for the vaccination.

### Relative Importance of Input Variables

The probability of contracting Lyme disease is the most important factor in determining the economic benefit of vaccinating against Lyme disease (Figure 2). The results from Figure 2 and from the sensitivity analyses concerning the costs of treating sequelae and vaccine effectiveness (Table 3) indicate that the next most important variables are the cost of treating sequelae and the probability of early detection and treatment of Lyme disease.

### Research Priorities

Given the importance of treatment costs in assessing the cost effectiveness of Lyme disease vaccine, accurate data regarding the cost of treating sequelae should receive high priority when setting a research agenda for Lyme disease. Data concerning the duration of the various forms of long-term sequelae and the indirect costs borne by patients are also important. For both items, research should not focus on obtaining a mean value but rather on collecting sufficient data to describe the probability distribution of these input variables, which could either replace the assumed distributions (Table 1) or be added to the model to further refine the results.

### Implications for Public Health Policy

Very few communities have an annual incidence of Lyme disease of 0.005 or higher. From 1992 to 1996, approximately 47% (1,483) of U.S. counties reported at least one case of Lyme disease. However, 148 counties (almost all in the northeastern and northcentral United States [CDC, unpub. data; 1]) reported 90.3% of cases. Connecticut and Rhode Island had the highest cumulative annual incidences of reported Lyme disease, equivalent to probabilities of contracting Lyme disease of 0.000949 and 0.000539, respectively (1996 data) (46). Two studies (47,48) have shown that cases have been underreported in areas where the disease is highly endemic. However, the range of probabilities in our model allows for both underreporting and overdiagnosis.

The benefits are likely highest if both community-level incidence of Lyme disease and individual risk for exposure to tick bites and infection (38) can be considered in using the vaccine. The Advisory Committee on Immunization Practices, Public Health Service, U.S. Department of Health and Human Services, recently agreed with this conclusion and voted, in February 1999, to recommend the use of Lyme disease vaccine on the basis of a combination of both community-level and individual risk. These recommendations will be published soon (49).

Ours is not the only study to suggest that the vaccine not be used universally. A forthcoming Institute of Medicine report (50) uses cost per quality-adjusted life year (QALY) saved to examine vaccine priorities. The authors estimate that it would cost more than $100,000 per QALY saved if the vaccine were given ". . . to resident infants born in, and immigrants of any age to, geographically defined high risk areas." This result led the authors to rank Lyme disease vaccine as "less favorable," their lowest ranking in terms of priorities for vaccine development.

Our model also considers the relative value of two interventions: vaccination and the detection and treatment of early Lyme disease. Communities with average individual probabilities of contracting Lyme disease of less than 0.01 may benefit from interventions that improve the probability of early diagnosis and treatment of Lyme disease.

Dr. Meltzer is senior health economist, Office of the Director, National Center for Infectious Diseases, CDC. His research interests focus on assessing the economics of public health interventions such as oral raccoon rabies vaccine, Lyme disease vaccine, and hepatitis A vaccine, as well as estimating the economic cost of bioterrorism, dengue, pandemic influenza, and other infectious diseases. His research uses various methods, including Monte Carlo modeling, willingness-to-pay surveys (contingent valuation), and the use of nonmonetary units of valuation, such as disability adjusted life years.

### References

1. Centers for Disease Control and Prevention. Lyme disease—United States, 1996. MMWR Morb Mortal Wkly Rep 1997;46:531-5.
2. Berglund J, Eitrem R, Ornstein K, Lindberg A, Ringner A. Elmrud J, et al. An epidemiologic study of Lyme disease in southern Sweden. N Engl J Med 1995;333:1319-24.
3. Strle F, Stantic-Pavlinic M. Lyme disease in Europe [letter, comment]. N Engl J Med 1996;334:803.
4. Cartter ML, Mshar P, Hadler JL. The epidemiology of Lyme disease in Connecticut. Conn Med 1989;53:320-3.
5. Ginsberg HS. Geographical spread of *Ixodes dammini* and *Borrelia burgdorferi*. In: Ginsberg HS, editor. Ecology and environmental management of Lyme disease. New Brunswick (NJ): Rutgers University Press; 1993. p. 63-82.
6. White DJ, Chong HG, Benach JL, Bosler EM, Meldrum SC, Means RG, et al. The geographic spread and temporal increase of the Lyme disease epidemic. JAMA 1991;266:1230-6.
7. Dennis DT. Lyme disease. Dermatol Clin 1995;13:537-51.
8. Schwartz BS, Goldstein MD, Childs JE. Antibodies to *Borrelia burgdorferi* and tick salivary gland proteins in New Jersey outdoor workers. Am J Public Health 1993;83:1746-8.
9. Steere AC, Sikand VK, Meurice F, Parenti DL, Fikrig E, Schoen RT, et al. Vaccination against Lyme disease with recombinant *Borrelia burgdorferi* outer-surface lipoprotein A with adjuvant. N Engl J Med 1998;339;209-15.
10. Sigal HL, Zahradnik JM, Levin P, Patella SJ, Bryant G, Haselby R, et al. A vaccine consisting of recombinant *Borrelia burgdorferi* outer-surface protein A to prevent Lyme disease. N Engl J Med 1998;339;216-22.
11. Centers for Disease Control and Prevention. Notice to readers: availability of Lyme disease vaccine. MMWR Morb Mortal Wkly Rep 1999;48:35-6,43.
12. Snider DE, Holtgrave DR, Duñet DO. Decision analysis. In: Haddix AC, Teutsch SM, Shaffer PA, Duñet DO, editors. Prevention effectiveness: a guide to decision analysis and economic evaluation. New York: Oxford University Press; 1996. p. 27-46.
13. Palisade Corporation. Guide to using @Risk (Windows version). Newfield (NY): Palisade Corporation; 1996.
14. Dittus RS, Roberts SD, Wilson JR. Quantifying uncertainty in medical decisions. J Am Coll Cardiol 1989;14:23A-8.
15. Critchfield GC, Willard KE. Probabilistic analysis of decision trees using Monte Carlo simulation. Med Decis Making 1986;6:85-92.
16. Dobilet P, Begg CB, Weinstein MC, Braun P, McNeil BJ. Probabilistic sensitivity analysis using Monte Carlo simulation: a practical approach. Med Decis Making 1985;5:157-77.

17. Telford SR, Kantor FS, Lobet Y, Barthold SW, Spielman A, Flavell RA, et al. Efficacy of human Lyme disease vaccine formulations in a mouse model. J Infect Dis 1995;171:1368-70.
18. De Silva AM, Telford SR III, Brunet LR, Barthold SW, Fikrig E. *Borrelia burgdorferi* OspA is an arthropod-specific transmission-blocking Lyme disease vaccine. J Exp Med 1996;183:271-5.
19. Straubinger RK, Chang YF, Jacobson RH, Appel MJG. Sera from OspA-vaccinated dogs, but not those from tick-infected dogs, inhibit in vitro growth of *Borrelia burgdorferi*. J Clin Microbiol 1995;33:2745-51.
20. Philipp MT, Lobet Y, Bohm RP, Conway MD, Dennis VA, Desmons P, et al. Safety and immunogenicity of recombinant outer surface protein A (OspA) vaccine formulations in the rhesus monkey. Journal of Spirochetal Tickborne Disease 1996:67-79.
21. Keller D, Koster FT, Marks DH, Hosbach P, Erdile LF, Mays JP. Safety and immunogenicity of a recombinant outer surface protein A Lyme Vaccine. JAMA 1994;271:1764-8.
22. Schoen RT, Meurice F, Brunet CM, Cretella S, Krause DS, Craft JE, et al. Safety and immunogenicity of an outer surface protein A vaccine in subjects with previous Lyme disease. J Infect Dis 1995;172:1324-9.
23. Padilla ML, Callister SM, Schell RF, Bryant GL, Jobe DA, Loverich SD, et al. Characterization of the protective borreliacidal antibody response in humans and hamsters after vaccination with a *Borrelia burgdorferi* outer surface protein A vaccine. J Infect Dis 1996;174:739-46.
24. Steere AC. Lyme disease. N Engl J Med 1989;321:586-96.
25. Massarotti EM, Luger SW, Rahn DW, Messner RP, Wong JB, Johnson RC, et al. Treatment of early Lyme disease. Am J Med 1992;92:396-403.
26. Luger SW, Paparone P, Wormser GP, Nadelman RB, Grunwaldt E, Gomez G, et al. Comparison of cefuroxime axetil and doxycycline in treatment of patients with early Lyme disease associated with erythema migrans. Antimicrob Agents Chemother 1995;39:661-7.
27. Dattwyler RJ, Luft BJ, Kunkel MJ, Finkel MF, Wormser GP, Rush TJ, et al. Ceftriaxone compared with doxycycline for the treatment of acute disseminated Lyme disease. N Engl J Med 1997;337:289-94.
28. Steere AC, Levin RE, Molloy PJ, Kalish RA, Abraham JH, Liu NY, et al. Treatment of Lyme arthritis. Arthritis Rheum 1994;37:878-88.
29. Magid D, Schwartz B, Craft J, Schwartz JS. Prevention of Lyme disease after tick bites: a cost-effectiveness analysis. N Engl J Med 1992;327:534-41.
30. Nichol G, Dennis DT, Steere AC, Lightfood R, Wells G, Shea B, et al. Test-treatment strategies for patients suspected of having Lyme disease: a cost-effectiveness analysis. Ann Intern Med 1998;128:37-48.
31. Lightfoot RW, Luft BJ, Rahn DW, Steere AC, Sigal LH, Zoschke DC, et al. Empiric parenteral antibiotic treatment of patients with fibromyalgia and fatigue and a positive serological result for Lyme disease. Ann Intern Med 1993;119:503-9.
32. Shadick NA, Phillips CB, Logigian EL, Steere AC, Kaplan RF, Berardi VP, et al. The long-term clinical outcomes of Lyme disease. Ann Intern Med 1994;121:560-7.
33. Alpert B, Esin J, Sivak SL, Wormser GP. Incidence and prevalence of Lyme disease in a suburban Westchester County community. New York State Journal of Medicine 1992;92:5-8.
34. Steere AC, Taylor E, Wilson ML, Levine JF, Spielman A. Longitudinal assessment of the clinical and epidemiological features of Lyme disease in a defined population. J Infect Dis 1986;154:295-300.
35. Kaslow RA, Samples CL, Simon DG, Lewis JN. Occurrence of erythema chronicum migrans and Lyme disease among children in two noncontiguous Connecticut counties. Arthritis Rheum 1981;24:1512-6.
36. Hanrahan JP, Benach JL, Coleman JL, Bosler EM, Morse DL, Cameron DJ, et al. Incidence and cumulative frequency of endemic Lyme disease in a community. J Infect Dis 1984;150:489-95.
37. Lastavica CC, Wilson ML, Berardi VP, Spielman A, Deblinger RD. Rapid emergence of a focal epidemic of Lyme disease in costal Massachusetts. N Engl J Med 1989;320:133-7.
38. Maes E, Lecomte P, Ray N. A cost-of-illness study of Lyme disease in the United States. Clin Ther 1998;20:993-1008.
39. Evans M, Hastings N, Peacock B. Statistical distributions. 2nd ed. New York: John Wiley; 1993.
40. Statistical abstract of the United States. 117th ed. Washington: U.S. Bureau of the Census; 1997.
41. Meltzer MI, Teutsch SM. Setting priorities for health needs, managing resources. In: Teutsch SM, Stroup DF, editors. Quantitative solutions to public health problems. New York: Oxford University Press; 1998. p. 123-49.
42. Haddix AC, Shaffer PA. Cost-effectiveness analysis. In: Haddix AC, Teutsch SM, Shaffer PA, Duñet DO, editors. Prevention effectiveness: a guide to decision analysis and economic evaluation. New York: Oxford University Press; 1996. p. 103-29.
43. The Federal Register. Vol 61; no. 170; 1996 Aug 30; 46301-2.
44. Productivity loss tables [Appendix I]. In: Haddix AC, Teutsch SM, Shaffer PA, Duñet DO, editors. Prevention effectiveness: a guide to decision analysis and economic evaluation. New York; Oxford University Press; 1996. p. 187-92.
45. Fix AD, Strickland T, Grant J. Tick bites and Lyme disease in an endemic setting. JAMA 1998;279:206-10.
46. Centers for Disease Control and Prevention. Lyme disease—United States, 1996. MMWR Morb Mortal Wkly Rep 1997;46:531-5.
47. Coyle BS, Strickland GT, Liang YY, Peña C, McCarter R, Israel E. The public health impact of Lyme disease in Maryland. J Infect Dis 1996;173:1260-2.
48. Meek JL, Roberts CL, Smith EV, Cartter ML. Underreporting of Lyme disease by Connecticut physicians, 1992. Journal of Public Health Management Practice 1996;2:61-5.
49. Centers for Disease Control and Prevention. Prevention of Lyme disease through active immunization: recommendations of the Advisory Committee on Immunization practices (ACIP). MMWR Morb Mortal Wkly Rep. In press 1999.
50. Stratton KR, Durch JS, Lawrence RS, editors. Vaccines for the 21st century: a tool for decision making. Washington: National Academy Press. In press 1999.
